# Advances in chimeric antigen receptor T-cell therapy for B-cell non-Hodgkin lymphoma

**DOI:** 10.1186/s40364-021-00309-5

**Published:** 2021-07-13

**Authors:** Zixun Yin, Ya Zhang, Xin Wang

**Affiliations:** 1 Department of Hematology, Shandong Provincial Hospital, Cheeloo College of Medicine, Shandong University, Jinan, 250021 Shandong China; 2 School of Medicine, Shandong University, Jinan, 250021 Shandong China; 3 Department of Hematology, Shandong Provincial Hospital Affiliated to Shandong First Medical University, Jinan, 250012 Shandong China; 4 Shandong Provincial Engineering Research Center of Lymphoma, Jinan, 250021 Shandong China; 5 Branch of National Clinical Research Center for Hematologic Diseases, Jinan, 250021 Shandong China; 6 National Clinical Research Center for Hematologic Diseases, the First Affiliated Hospital of Soochow University, Suzhou, 251006 China

**Keywords:** Chimeric antigen receptor T-cell therapy, CAR-T-associated toxicities, New targets

## Abstract

B-cell non-Hodgkin lymphoma (B-NHL) is a group of heterogeneous disease which remains incurable despite developments of standard chemotherapy regimens and new therapeutic agents in decades. Some individuals could have promising response to standard therapy while others are unresponsive to standard chemotherapy or relapse after autologous hematopoietic stem-cell transplantation (ASCT), which indicates the necessity to develop novel therapies for refractory or relapsed B-NHLs. In recent years, a novel cell therapy, chimeric antigen receptor T-cell therapy (CAR-T), was invented to overcome the limitation of traditional treatments. Patients with aggressive B-NHL are considered for CAR-T cell therapy when they have progressive lymphoma after second-line chemotherapy, relapse after ASCT, or require a third-line therapy. Clinical trials of anti-CD19 CAR-T cell therapy have manifested encouraging efficacy in refractory or relapsed B-NHL. However, adverse effects of this cellular therapy including cytokine release syndrome, neurotoxicity, tumor lysis syndrome and on-target, off-tumor toxicities should attract our enough attention despite the great anti-tumor effects of CAR-T cell therapy. Although CAR-T cell therapy has shown remarkable results in patients with B-NHL, the outcomes of patients with B-NHL were inferior to patients with acute lymphoblastic leukemia. The inferior response rate may be associated with physical barrier of lymphoma, tumor microenvironment and low quality of CAR-T cells manufactured from B-NHL patients. Besides, some patients relapsed after anti-CD19 CAR-T cell therapy, which possibly were due to limited CAR-T cells persistence, CD19 antigen escape or antigen down-regulation. Quite a few new antigen-targeted CAR-T products and new-generation CAR-T, for example, CD20-targeted CAR-T, CD79b-targeted CAR-T, CD37-targeted CAR-T, multi-antigen-targeted CAR-T, armored CAR-T and four-generation CAR-T are developing rapidly to figure out these deficiencies.

## Background

**Fig. 1:**
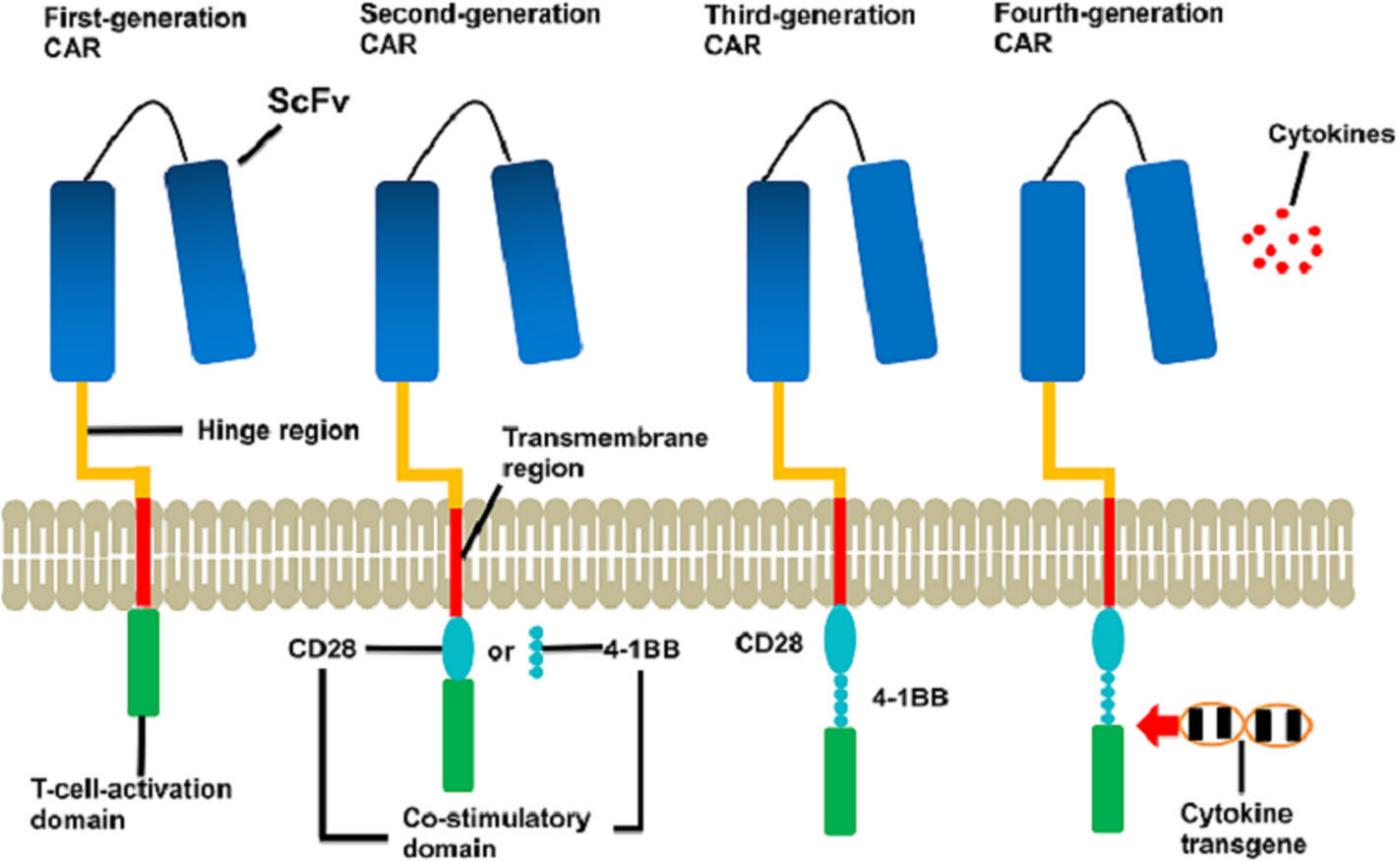
The structure of four generations of chimeric antigen receptor (CAR). Chimeric antigen receptor contains a single-chain variable region (scFv) of an antibody, hinge region, transmembrane region, co-stimulatory domain (except first-generation CAR) and a T-cell-activation domain. First-generation CAR has a single signaling molecule such as CD3ζ and lacks a co-stimulatory domain such as CD28 or 4-1BB. The second-generation CAR has a co-stimulatory domain derived from either CD28 or 4-1BB and can generate double signals via costimulatory domain to enhance the activity and persistence of CAR-T cell in vivo. Third-generation CAR has two costimulatory domains such as CD28 and 4-1BB. Fourth-generation CAR can produce and release transduced cytokines to enhance the activity of CAR-T cells and change the tumor microenvironment

**Fig. 2:**
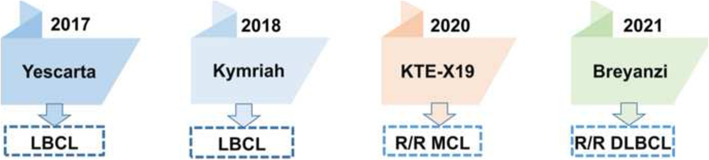
Four CAR-T cell products have been approved by the United States Food and Drug Administration (FDA): Yescarta for large B-cell lymphoma (LBCL) such as diffuse large B-cell lymphoma (DLBCL), transformed follicular lymphoma and primary mediastinal B-cell Lymphoma in 2017. Kymriah for LBCL in 2018. Then brexucabtagene autoleucel (KTE-X19) was approved by FDA for refractory / relapsed mantle cell lymphoma (R/R MCL) in 2020. In 2021, Breyanzi (liso-cel) was approval by FDA for patients with refractory / relapsed DLBCL (R/R DLBCL)

B-cell non-Hodgkin lymphoma (B-NHL) is a group of clinically heterogeneous disease including diffuse large B-cell lymphoma (DLBCL), mantle cell lymphoma (MCL), follicular lymphoma (FL) and others [1]. DLBCL is the most common kind of B-NHL, accounting for about 30–35% in all B-NHLs [2]. The standard first-line therapy is cyclophosphamide, doxorubicin, vincristine, and prednisone, with (R-CHOP) or without rituximab (CHOP). The second-line therapies consist of high-dose chemotherapy and autologous hematopoietic stem-cell transplantation (ASCT) [3]. These standard therapies have improved outcomes of patients with B-NHL. However, the outcomes of chemotherapy-refractory patients remained poor and almost one third of patients with DLBCL relapsed [4, 5]. CAR-T therapy is a novel treatment for these patients with refractory or relapsed lymphoma or acute lymphoblastic leukemia (ALL) in recent years. Many clinical trials have demonstrated the efficacy of CAR-T cell therapy against B-cell lymphoma. CARs are artificial proteins containing antigen recognition domains, T-cell signaling domains and other components. Most CARs consist of four parts. Extracellular targeting domains are usually single chain variable fragments (scFv), which composes the variable regions of heavy and light chains in antibodies [6]. A hinge or spacer, which is designed to connect scFv with transmembrane domain. Transmembrane domain is designed to assist scFv and intracellular signaling domain. And intracellular signaling domains, including co-stimolatory domain and T-cell activation domain, play roles on the activation and proliferation of CAR-T cells (Fig. 1) [5]. Four CAR-T cell products have been approved by the United States Food and Drug Administration (FDA) and/or European Medicines Agency (EMA): axicabtagene ciloleucel (axi-cel) for patients with refractory large B-cell lymphoma in 2017, tisagenlecleucel (tisa-cel) for refractory/relapsed ALL in 2017 and refractory/relapsed DLBCL in May 2018, brexucabtagene autoleucel (KTE-X19) for patients with refractory/relapsed MCL in 2020 and breyanzi (liso-cel) for patients with refractory/relapsed DLBCL in 2021 (Table 1; Fig. 2) [7]. Patients with aggressive B-cell lymphoma are considered for anti-CD19 CAR-T when they have stable or progressive lymphoma after second-line chemotherapy, relapse after ASCT, or require a third-line therapy [8]. In this review, we discuss CAR-T clinical trials on B-NHL, adverse events, limitations and new developments of CAR-T cell therapy.

**Table 1:** The approved CAR-T products for B-cell non-Hodgkin lymphoma

Product	CAR construct	Year of approval	Indication	Bridging therapy	CAR-T dose	ORR	CR rate	Median PFS	Incidence of grade 3/4 CRS	Incidence of grade3/4 ICANS
Axicabtagene ciloleucel	CD19-CD28-CD3ζ	2017	Refractory LBCL	No	2 × 10^6 cell/kg	83%	58%	5.9 months (3.3 to 15.0)	11% (12/108)	32%
Tisagenlecleucel	CD19–4-1BB - CD3ζ	2018	Relapsed or refractory DLBCL in adults	Yes	(0.1–6) × 10^8 CAR-positive viable T cells	52%	40%	8.3 months (5.8 to 11.7)	22%	12%
Brexucabtagene autoleucel	CD19-CD28-CD3ζ	2020	Relapsed or refractory MCL	Yes	2 × 10^6 cell/kg	85%	59%	4.8 months	15%	31%
Breyanzi (Lisocabtagene maraleucel)	CD19–4-1BB - CD3ζ	2021	Relapsed or refractory LBCL	Yes	50 × 10^6^, 100 × 10^6^ and 150 × 10^6^ CAR-positive T cells	73%	53%	NR	2% (6/269)	10% (27/269)

Abbreviations: *ORR* objective response rate, *CR* complete response, *PFS* progression-free survival, *CRS* cytokine release syndrome, *ICANS* immune effector cell-associated neurotoxicity syndrome, *LBCL* large B-cell lymphoma, *DLBCL* diffuse large B-celllymphoma, *MCL* mantle-cell lymphoma

### Clinical trials of anti-CD19 CAR-T in B-NHL

The antigen CD19 is widely expressed on B-cell malignancies including a wide range of B-NHL. Therefore, CD19 is an optimal target for CAR-T cell therapy in B-NHL. Anti-CD19 CAR-T cells have become the most widely applied CAR-T products and achieved encouraging improvements in clinical practice. The first case report of CD19 CAR-T therapy for one patient with B-cell lymphoma was reported in 2010. There were several completed CAR-T clinical trails for B-NHL (Table 2). For instance, NCT03483688 was a phase I b study which evaluated the safety and efficacy of C-CAR011, a CD19-directed CAR-T cells, in 6 patients. NCT02132624 was a phase I/II a study which evaluated the efficacy and safety of CD19-targeted third-generation CAR-T cells for refractory B-cell malignancies including B-cell lymphoma and B-cell leukemia in 15 individuals [9–12]. Besides, many CAR-T phase I and phase II clinical trials for pediatric and adult’s B-cell lymphoma are under recruiting status and the majority of them are anti-CD19 CAR-T trials (Table 3) [13]. Moreover, many clinical trials are ongoing and this review will introduce some of these trials.

**Table 2:** Completed CD19 CAR-T clinical trials for B-cell non-Hodgkin lymphoma

NCT Number	Phase	Status	Locations	Clinical outcomes
NCT01593696	Phase I	Completed	National Institutes of Health Clinical Center, 9000 RockvillePike, Bethesda, Maryland, United States	**The CR rate was 59.6% (31/52). The occurrence rate of serious adverse events and high grade CRS were 26.9% (14/52) and 9.6% (5/52).**
NCT02132624	Phase I / IIa	Completed	Uppsala University Hospital, Dept of Oncology, Uppsala,Sweden	Six of the 15 patients had complete responses (4/11 lymphoma and 2/4 ALL). Three patients developed severe CRS in 15 patients and 2 patients developed high grade ICANS.
NCT02030834	Phase IIa	Completed	Abramson Cancer Center of the University of Pennsylvania,Philadelphia, Pennsylvania, United States	The objective response rate was 64% (18/28). Occurrence rate of serious CRS and ICANS were 18% (5/28) and 11% (3/28)
NCT01626495	Phase I /IIa	Completed	CHOP-http://www.chop.edu/service/oncology/pediatric-cancer-research/cart-19-trial.html, Philadelphia, Pennsylvania, UnitedStates	The CR rate was 25.8% (16/62) and CRi (complete remission with incomplete blood count recovery) rate was 61.3% (38/62).

Abbreviations: *CR* complete response, *CRS* cytokine release syndrome, *ICANS* immune effector cell-associated neurotoxicity syndrome, *CRi* complete remission with incomplete blood count recovery, *ALL* acute lymphoblastic leukemia

**Table 3:** Clinical trials of chimeric antigen receptor T-cell therapy for B-cell lymphoma

NCT Numberb	Conditionso	Interventiont	P Phase h	Number	Dose D	Outcomes O
NCT04169932	R/R B-cell lymphoma; NHL	CD20 CAR-T	Early phase I	20	(1, 2, 4, 8) **×** 10^6 cells/kg	NR
NCT03994913	R/R B-NHL	CD19 CAR-T	Phase I Phase II	78	NR	NR
NCT04036019	B-cell lymphoma	CD20 CAR- T	Phase I	12	NR	NR
NCT04429438	B-cell lymphoma	4SCAR19 and 4SCAR20/22/70/PSMA/13/79b/GD2	Phase I Phase II	11	NR	NR
NCT04381741	DLBCL	CD19 CAR-T plus PD1 monoclonal antibody	Phase I	24	(1, 2, 3)**×** 10^6 cells/kg plus 200 mg Tislelizumab every 3 weeks for 6 times	NR
NCT04260932	B-cell lymphoma	CD19/CD20 Dual- CAR-T	Phase I	12	Escalated dose of (1–6) **×** 10^6 cells/kg	NR
NCT03929107	B-cell lymphoma	IL-7 and chemokine ligand 19-expressing CD19-CAR-T cells	Phase II	80	NR	NR
NCT03854994	B-cell lymphoma; B-ALL	CD19 CAR-T Cells	Phase I	10	(1–5) **×** 10^6 cells/kg	NR
NCT04316624	DLBCL	C-CAR066	Phase I	10	Low: (1.0–3.0) **×** 10^6 cells/kg; Medium: (3.0–6.0) **× 10^6 cells/kg;** **High: (6.0–9.0) × 10^6 cells/kg**	NR
NCT04240808	NHL	UCD19 CAR T Cells	Phase I	20	NR	NR
NCT04539444	R/R NHL	CD19/22 CART	Phase II	20	3-day split-dose regimen at dose of (0.5–2) **× 10^6 cells/kg**	NR
NCT03881761	B-cell lymphoma	CD19/CD20 bispecific CAR-T	Phase I	50	(1–3) **× 10^6 cells/kg**	NR
NCT04486872	DLBCL	Autologous humanized CD19/CD20 bispecific CAR-T	Phase I	18	1.00 **× 10^6 cells/kg, 3.00 × 10^6 cells/kg or 5.00 × 10^6 cells/kg**	NR
NCT04257578	B-NHL; DLBCL; High grade B-cell lymphoma; PMBCL; tFL	Axicabtagene Ciloleucel	Phase I Phase II	20	NR	NR
NCT03391726	B-cell lymphoma	CD19 CAR-T	Phase II	20	NR	NR
NCT04416984	B-cell lymphoma	ALLO-647	Phase I Phase II	120	NR	NR
NCT03103971	B-ALL; DLBCL; PMBL; R/R B-NHL	CD19 CAR-T	Phase I	73	NR	NR
NCT03287817	DLBCL; R/R DLBCL	AUTO3	Phase I Phase II	171	(50–900) **× 10^6 cells**	NR
NCT04049513	B-NHL; DLBCL; PMBCL; tFL; FL; MCL	WZTL002–1	Phase I	12	Starting dose of 5 **× 10^4 cells/kg**	NR
NCT04432506	R/R DLBCL; R/R high grade B-cell lymphoma; R/R PMBCL; R/R tFL	Axicabtagene Ciloleucel	Phase I	20	NR	NR
NCT04532268	ALL; B-NHL	Humanized CD19 CAR-T cells	Early phase I	72	NR	NR
NCT03932955	Lymphoma	MC-19PD1 CAR-T cells	Phase I	15	NR	NR
NCT04191941	B-NHL; B-ALL; MM	Novel CAR-T	Phase I	9	NR	NR
NCT04007029	CLL; R/R DLBCL; FL; MCL; PMBCL; SLL	CAR-T	Phase I	24	NR	NR
NCT04163302	B-cell lymphoma	CD19-PD1-CAR-T Cell	Phase II	30	NR	NR
NCT04215016	R/R DLBCL Patients With Either CD19 or CD20 Positive	Autologous humanized CD19/CD20 bispecific CAR-T	Phase I	18	The first dose is 1.0 **× 10^6 cells/kg, the second dose is 3.0 × 10^6 cells/kg and the third dose is 8.0 × 10^6/kg.**	NR
NCT03664635	R/R B-NHL	MB-CART20.1	Phase I Phase II	19	Safety dose level: 1 **× 10^5 cells/kg;** **Dose level 1: 1 × 10^6 cells/kg;** **Dose level 2: 3 × 10^6 cells/kg.**	NR
NCT04088890	B-ALL; B-NHL; DLBCL	CD22 CAR-T	Phase I	95	ALL: 3 **× 10^5 cells/kg(±20%); B-NHL: 1 × 10^6 cells/kg, 3 × 10^6 cells/kg and 1 × 10^7 cells/kg**	NR
NCT03366324	ALL; B-cell lymphoma	Second generation CAR-T cells	Phase I Phase II	20	NR	NR
NCT03277729	R/R B-NHL; CLL; R/R DLBCL; Recurrent FL; Recurrent MCL; Recurrent MZL	CAR-T	Phase I Phase II	30	NR	NR
NCT04532281	ALL; NHL	CD19 CAR-T	Early phase I	120	NR	NR
NCT03398967	B-cell lymphoma	Dual specificity CD19 and CD20 or CD22 CAR-T Cells	Phase I Phase II	80	NR	NR
NCT02965092	ALL; B-cell lymphoma	Second generation CAR-T cells	Phase I Phase II	80	(0.89–4.01) **×** 10^6 CAR-T cells/kg	Median overall survival (OS) was 16.1 months. The 6-month and 12-month OS rates were 69.727 and 64.028%.
NCT02963038	B-cell lymphoma	Autologous CD19- targeting CAR T	Phase I Phase II	10	NR	NR
NCT03383952	B-cell lymphoma	ICAR19 CAR-T cells	Phase I	20	NR	NR
NCT04007978	B-cell lymphoma; ALL	Third generation CAR- T	Phase I	50	NR	NR
NCT04532203	ALL; NHL	CAR-T cells	Early phase I	72	NR	NR
NCT04603872	R/R MM; R/R ALL; R/R NHL	CD19/BCMA CAR T-cells	Early phase I	120	NR	NR
NCT03939026	R/R Large B-Cell Lymphoma; R/R FL	ALLO-647	Phase I	54	NR	NR
NCT04008251	ALL; B-cell lymphoma	Second generation humanized CAR-T	Phase I	10	NR	NR
NCT03118180	Lymphoma	CD19 CAR-T	Phase I Phase II	50	NR	NR
NCT02650414	B-cell lymphoma	CD22 CAR-T	Phase I	15	Subjects < 50 kg: (0.2–1) **× 10^7 cells/kg** Subjects > 50 kg: (1–5) **× 10^8 cells**	NR
NCT04317885	B-NHL	CD19/CD20-directed CAR-T cells	Phase I	25	NR	NR
NCT03166878	B-cell lymphoma	UCART019	Phase I Phase II	80	Day 0: 10% of total dose. Day 1: 30% of total dose if patient is stable. Day 2: 60% of total dose if patient is stable.	NR
NCT03720457	DLBCL; FL	CD19 CAR-T	Phase I	18	NR	NR
NCT03559439	B-cell lymphoma; B-ALL	CD19 CAR T	Phase I	9	Low: 1 **× 10^5 cells/kg; Medium: 2 × 10^6 cells/kg; High: 6 × 10^6 cells/kg**	NR
NCT04088864	B-cell lymphoma; ALL	Autologous CD22 CAR T	Phase I	52	R/R B-ALL: 1 **× 10^6 cells/kg (±20%).** **Lymphoma: 1 × 10^6 cells/kg (±20%).**	NR
NCT03696784	B-cell lymphoma	iC9-CAR19 T cells	Phase I	30	(1–2) **× 10^6 cells/kg**	NR
NCT04204161	R/R ALL; B-cell lymphoma	CAR-T19/CAR-T22	Phase I	30	The recommand dose: 1 **×** 10^5/kg-2.5 **×** 10^8/kg	NR
NCT03853616	ALL; B-cell lymphoma; R/R CLL	MB-CART19.1	Phase I Phase II	48	1 **×** 10^5/kg-3 **×** 10^6/kg	NR
NCT03881774	B-cell lymphoma	CAR-T cells	Phase I	20	(0.5–3) **×** 10^6/kg	NR
NCT04176913	DLBCL; FL; MCL; SLL	LUCAR-20S CAR-T cells	Phase I	41	NR	NR
NCT04156243	B-cell lymphoma	CD19 CAR T cells	Early phase I	20	NR	NR
NCT04089215	NHL; DLBCL; FL	CD19 CAR-T	Phase II	82	1.0 **×** 10^8 and 1.5 **×** 10^8 cells	Three month ORR: 60.3%
NCT04205838	DLBCL; High grade B-cell lymphoma; R/R tFL; R/R PMBCL	Axicabtagene Ciloleucel	Phase II	36	NR	NR
NCT04464200	DLBCL; PMBCL; tFL; CLL; MZL; WM; Burkitt’s lymphoma; Primary CNS lymphoma	19(T2)28z1xx CAR T cells	Phase I	60	Planned flat-dose levels: (25, 50, 100, 150, 200) **×** 10^6 cells; De-escalation dose: 12.5 **×** 10^6 cells.	NR
NCT04545762	Refractory NHL; Burkitt lymphoma; MCL; FL; PMBCL; DLBCL; SLL	CD19 CAR-T cells	Phase I	36	Starting dose: 5 **×** 10^5 cells/kg	NR
NCT03999697	DLBCL; FL; MCL; Plasma cell neoplasm	CD22 CAR-T	Phase I	10	NR	NR
NCT04214886	B-ALL; B-cell lymphoma	CD19-CD34 CAR-T	Phase I	24	1 **×** 10^6 cells/kg (± 20%) to 2 **×** 10^6 cells/kg	NR
NCT03666000	NHL; B-ALL	PBCAR0191	Phase I Phase II	92	Starting dose: 3 **×** 10^5 cells/kg; Subsequent dose groups: escalating doses to a maximum dose of 9 **×** 10^6 cells/kg.	NR
NCT02772198	B-ALL; B-NHL	CD19 CAR T cells	Phase I Phase II	300	NR	ORR was 84% (30/36) in ALL and 62% (32/52) in NHL.
NCT04156178	B-cell lymphoma	CD20-CD19 CAR-T	Early phase I	12	NR	NR
NCT03743246	Precursor cell lymphoblastic leukemia- lymphoma; B-cell lymphoma	JCAR017	Phase I Phase II	121	0.05 **×** 10^6–0.75 **×** 10^6 cells/kg	NR
NCT03676504	ALL; CLL; DLBCL; FL; MCL	CD19 CAR T Cells	Phase I Phase II	48	1 **×** 10^6 cells/m^2 to 20 **×** 10^7 cells/m^2	NR
NCT03448393	ALL; B-cell lymphoma; B-NHL	CD19/CD22 CAR T- Cells	Phase I	89	1 **×** 10^5 cells/kg (+/− 20%); 3 **×** 10^5 cells/kg (+/− 20%); 1 **×** 10^6 cells/kg; 3 **×** 10^6 cells/kg (+/− 20%); 1 **×** 10^7 cells/kg (+/− 20%).	NR
NCT04186520	B-NHL; MCL	CAR-T	Phase I Phase II	32	2.5 **×** 10^6 cells/kg	NR
NCT02631044	NHL; DLBCL; FL; MCL; PMBCL	JCAR017	Phase I	314	50 **×** 10 CAR-T cells [one or two doses], 100 **×** 10 CAR-T cells, and 150 **×** 10 CAR-T cells.	ORR was 73% and CR rate was 53% among 256 patients with B-NHL.
NCT03744676	B-NHL; B-cell lymphoma; DLBCL	lisocabtagene maraleucel	Phase II	80	NR	NR
NCT02706405	DLBCL; PMBCL	JCAR014	Phase I	42	NR	NR
NCT04226989	ALL; NHL	CT-RD06	Early phase I	72	NR	NR
NCT04314843	B-cell lymphoma	Axicabtagene Ciloleucel	Phase I Phase II	36	NR	NR
NCT03497533	B-NHL	TriCAR-T-CD19	Phase I Phase II	6	(0.5–1) **×** 10^6 CAR-T cells/kg	NR
NCT03720496	B-NHL	CD19-TriCAR-T	Phase I	6	(0.1–1) **×** 10^6 cells/kg	NR
NCT03105336	FL; MZL; Indolent NHL	Axicabtagene ciloleucel	Phase II	160	NR	NR
NCT03310619	NHL; DLBCL; FL	JCAR017	Phase I Phase II	75	50 **×** 10^6 CAR-T cells; 100 **×** 10^6 CAR-T cells.	NR

Abbreviations: *CAR* chimeric antigen receptor, *ORR* objective response rate, *CR* complete response, *DLBCL* diffuse large B-cell lymphoma, *B-ALL* B-cell acute lymphoblastic leukemia, *NHL* non-Hodgkin lymphoma, *B-NHL* B-cell non-Hodgkin lymphoma, *R/R B-NHL* refractory/relapsed B-cell non-Hodgkin lymphoma, *PMBCL* primary mediastinal large B-cell lymphoma, *FL* follicular lymphoma, *tFL* transformed follicular lymphoma, *R/R tFL* refractory/relapsed transformed follicular lymphoma, *MCL* mantle cell lymphoma, *MM* multiple myeloma, *SLL* small lymphocytic lymphoma, *CLL* chronic lymphocytic leukemia, *MZL* marginal zone lymphoma, *WM* Waldenstrom macroglobulinemia

### KTE-X19

KTE-X19 in relapsed or refractory MCL: MCL is a CD5+ B-NHL with high heterogeneity from indolent to highly aggressive clinical course [14, 15]**.** Patients with MCL have unsatisfactory prognosis after standard treatments [16]. KTE-X19 is an anti-CD19 CAR-T cell therapy designed for patients with relapsed or refractory MCL. ZUMA-2 trial was a phase II, multicenter clinical trial to evaluate the efficacy and safety of KTE-X19. Seventy-four patients were enrolled in the trial. KTE-X19 was successfully manufactured for 71 patients and infused to 68 patients at a dose of 2 × 10^6 cells/kg. Conditioning chemotherapy with fludarabine and cyclophosphamide was administered before infusion of KTE-X19 [17]. Ninety-three percent of patients reached objective response (OR) and 67% of patients reached complete response (CR) among the first 60 treated patients. In all 74 patients, the objective response rate (ORR) was 85% and the CR rate was 59% with the median follow-up of 12.4 months. The estimated rate of progression-free survival (PFS) was 61% and overall survival rate was 83% at 12 months. All 68 patients developed at least one adverse event. The most common adverse event was pyrexia, which occurred in 94% (64/68) of patients. The cytokine release syndrome (CRS) occurred in 61 patients. Most CRS events were grade 1 or 2. Besides, 63% of patients experienced neurologic events, with 32% having grade 3 or higher neurologic events [17].

### KTE-C19

DLBCL is the most common subtype of non-Hodgkin lymphoma which accounts for almost 30% of B-NHL [18]. The multicenter ZUMA-1 phase I study evaluated the efficacy and safety of KTE-C19, which was autologous CD3ζ/CD28-based CAR-T cell therapy. Seven patients received conditioning chemotherapy with fludarabine (30 mg/m^2^) and cyclophosphamide (500 mg/m^2^) for 3 days before infusion of KTE-C19 at a dose of 2 × 10^6 cells/kg. The ORR was 71% (5/7) and the CR rate was 57% (4/7). Among all seven patients, 3 patients remained in CR at 12 months and CAR-T cells could be detected. One patient had a dose-limiting toxicity of grade 4 neurotoxicity and CRS, then the patient died of systematic inflammation. One patient experienced grade 3 or higher CRS events and four patients developed grade 3 or higher neurotoxicities. These symptoms disappeared within 1 month [19].

### Axicabtagene ciloleucel

Axicabtagene ciloleucel was one kind of autologous anti-CD19 CAR-T cell which consisted of CD28 transmembrane domain and CD28 costimulatory domain for patients with B-cell lymphoma. ZUMA-1, a multicenter, phase II trial in which 111 patients with DLBCL, primary mediastinal B-cell lymphoma, or transformed FL were enrolled. Some patients were refractory to previous therapies, and some patients relapsed after ASCT. In contrast to KTE-X19 and KTE-C19, patients did not receive systemic bridging chemotherapy before infusion of CAR-T cells. A total of 101 patients received infusion of axi-cel at a dose of 2 × 10^6 cells/kg. In 77 patients with DLBCL, 38 patients achieved CR and 25 patients reached partial response (PR). In other 24 patients with primary mediastinal B-cell lymphoma or transformed FL, 17 patients reached CR and 3 patients achieved PR at a minimum of 6 months of follow-up [20]. The ORR was 82 and 54% of patients achieved CR. Grade 3 or higher adverse events accounted for almost 95% in all adverse events. The most common symptoms were pyrexia (85% of patients). CRS occurred in 94 patients (93% of patients) and most were grade 1 or 2. Sixty-five patients (64% of patients) developed neurotoxicity and the majority of these patients developed grade 1 or 2 neurologic events. A total of 101 patients were followed up for a median of 27.1 months. The ORR was 83% and the CR rate was 58%. The incidence of adverse events was similar to that in previous reports. Grade 3 or higher CRS occurred in 12 patients (11% of patients) and 35 patients (32% of patients) developed grade 3 or higher neurotoxicity [21]. Besides, clinical trials of axicabtagene ciloleucel for patients with FL have demonstrated encouraging results recently. Eighty patients with FL were enrolled in the clinical trial. The ORR was 95% and CR rate was 81%. Grade 3 or higher CRS occurred in 7% of the patients and the incidence of grade 3 or higher neurologic adverse events was 15%.

### Hu19-CD828Z

Clinical trial of FMC63-28Z had shown that neurologic toxicity was a more vital clinical problem than CRS [22]. Hu19-CD828Z, a new developed anti-CD19 CAR, was designed with a scFv from a fully-human anti-CD19 monoclonal antibody. FMC63-28Z (axi-cel), however, had the murine scFv [23]. The clinical trial evaluated the efficacy and safety of Hu19-CD828Z. Patients received conditioning chemotherapy with fludarabine (30 mg/m^2^) and cyclophosphamide (500 mg/m^2^) for 3 days. Then they were administered with Hu19-CD828Z at 3 target doses: 0.66 × 10^6, 2 × 10^6 and 6 × 10^6 cells/kg. Twenty patients with B-cell lymphoma who received previous treatments were enrolled in the trial. Incidence of neurologic events in clinical trial of Hu19-CD828Z was lower than that in clinical trial of FMC63-28Z. The occurrence of grade 2 or higher neurotoxicity of Hu19-CD828Z and FMC63-28Z were 20 and 77%, respectively. Besides, the incidence of grade 3 or higher neurologic events of Hu19-CD828Z and FMC63-28Z were 5 and 50%, respectively. These results may indicate the higher safety of Hu19-CD828Z. Furthermore, serum levels of immunological proteins such as IL-2, TNF-α and IFN-γ were lower in patients who received Hu19-CD828Z T cells than that in patients who received FMC63-28Z T cells. Naive and central memory (CM) T cells had more proliferation potential than effector memory and T-effector memory T cells. And it was confirmed that there was a higher percentage of CD4+ CAR-T cells with naive or CM in Hu19-CD828Z T cells than those in FMC63-28Z. In conclusion, patients treated with Hu19-CD828Z had lower incidence of developing neurologic events, lower levels of immunological proteins in serum, higher proliferation potentials of T cells and longer-term persistence of CAR-T cells [24].

### Tisagenlecleucel

There was a multicenter, international phase II study of tisagenlecleucel involving adult patients who cannot receive ASCT for some reasons or had poor prognosis after ASCT [25]. Tisagenlecleucel consisted of CD8 transmembrane domain and 4-1BB costimulatory domain. In this clinical trial, 165 patients were enrolled and 111 received an infusion of tisagenlecleucel at a median dose of 3.0 × 10^8 T cells (range 0.1 × 10^8 to 6.0 × 10^8). Ninety-two percent of the patients received bridging therapy before infusion of CAR-T cells. Among 93 patients who had 3 months or more follow-up, the best ORR was 52%. Forty percent of patients received CR and 12% of patients reached PR. Thirty-eight percent of patients achieved a response and the CR rate was 32% at 3 months follow-up. Thirty percent of patients remained in CR at 6 months. At 12 months follow-up, the overall survival rate was 49% among all patients who received infusion of CAR-T cells and 95% of those patients still remained in CR**.** The most common adverse events was CRS, which occurred in 58% of patients. Fourteen percent of patients received tocilizumab and 10% of patients received both tocilizumab and glucocorticoids for CRS management. Neurologic events occurred in 21% of patients. Among these patients, nine patients had grade 3 or 4 neurologic events and CRS at the same time [25].

### Lisocabtagene maraleucel

Lisocabtagene maraleucel is an autologous anti-CD19 CAR-T cell product with a CD28 transmembrane domain and 4-1BB co-stimulatory domain for patients with relapsed or refractory large B-cell lymphoma. Lisocabtagene maraleucel had a defined 1:1 ratio of CD4:CD8 T cells. There was a multicentre study of lisocabtagene maraleucel, named as TRANSCEND, to evaluate the efficacy and safety of lisocabtagene maraleucel. A total of 344 patients were enrolled. Fifty patients failed to receive CAR-T cells for some reasons among them. Fifty-nine percent of the patients received bridging therapy before infusion. Patients were assigned to one of three target dose levels (50 × 10^6^, 100 × 10^6^and 150 × 10^6^ CAR-T cells). Twenty-five patients received non-conforming CAR-T cells and 269 patients succeeded in infusion of at least one dose of CAR-T cells among the remaining 294 patients. However, thirteen patients were excluded from 269 patients for no PET-positive disease. In the end, there were 256 patients included in the efficacy-evaluable set. One hundred and eighty-six (73%) patients reached OR, with 53% of patients in CR. CRS occurred in 113 patients (42%) and grade 3 or 4 CRS occurred in 6 (2%) patients. Moreover, 27 (10%) patients experienced grade 3 or 4 neurotoxicities [26].

## Discussion of clinical trials

Clinical trials of CAR-T have demonstrated durable remission. However, the response rates were different. The construct of CAR-T product may contribute to the difference in response rates. Axicabtagene ciloleucel consists of CD28 transmembrane domain and CD28 co-stimulatory domain [20]. Tisagenlecleucel consists of CD8 transmembrane domain and 4-1BB co-stimulatory domain [25]. And lisocabtagene maraleucel is composed of CD28 transmembrane domain and 4-1BB co-stimulatory domain. The ORR was 82, 52 and 73%, respectively. Patients who received CAR-T products with CD28 transmembrane domains tended to have higher ORR than patients who received CAR-T products with CD8 transmembrane domains in clinical trials. Besides, the bridging therapy before infusion could affectresponse rates. Patients did not receive systemic bridging therapy in ZUMA-1. The ORR and CR rate were 82 and 54%, respectively. In clinical trial of tisagenlecleucel, most patients received bridging therapy. The ORR and CR rate were lower than that in ZUMA-1. There was one clinical study to evaluate the impact of bridging therapy on outcomes of 148 patients with B-NHL who underwent leukapheresis for axicabtagene ciloleucel infusion. The one-year PFS rate was 40% and the overall survival rate was 65% in patients who did not receive bridging therapy. These two rates were 21 and 48% in patients who received bridging therapy [27]. However, the reasons why bridging therapy could have impact on response and survival rate in B-NHL patients treated with CAR-T cell therapy are still unclear. Moreover, host systematic inflammation and tumor burden before treatment can be determining factors for the clinical outcomes of CAR-T cell therapy [28].

### Chimeric antigen receptor T-cell-associated toxicities and managements

Despite the remarkable clinical efficacy for patients with B-NHL or other B-cell malignancies, CAR-T cell-associated toxicities such as CRS can be observed in clinical trials, which limit the number of patients who are eligible for CAR-T cell therapy. CRS and immune effector cell-associated neurotoxicity syndrome (ICANS) are the two most common CAR-T cell-associated toxicities [29].

### CRS

CRS is the most common adverse event of CAR-T cell therapy [30]. CRS is defined as a clinical syndrome that may occur after cell therapy due to the release of cytokines (substances secreted by immune cells) into the body’s blood stream. The common clinical manifestations of CRS include fever, myalgias, headache, fatigue or other ful-like clinical presentations. Hypotension, hypoxia, coagulopathy and multiorgan dysfunction are severe symptoms of CRS. Severe symptoms are life-threatening and patients often need vasopressors, ventilation support and other supportive treatments [31, 32]. Fever occurs firstly in the majority of patients and serves as the hallmark of CRS while the severity of CRS is usually judged by hypotension and hypoxia [33]. Abnormal indexes detected in patients with CRS include elevated C-reactive protein (CRP), ferritin, interleukin-6 (IL-6), tumor necrosis factor alpha (TNFα), interferon gamma (IFNγ), cytopenias, coagulation abnormalities, elevated liver function, and elevated creatinine. Analysis of clinical data showed that high tumor burden, high T-cell doses and high peak of T-cell expansion can increase the incidence of CRS. The severity of CRS may be associated with tumor burden, inflammatory levels, conditioning therapy and doses of CAR-T cells. CRS is triggered by inflammatory cytokines and chemokines released by CAR-T cells. The immune process begins with CAR-T recognizing and binding to targeted cells, then the activated CAR-T cells can produce effector cytokines such as IFNγ, TNFα and IL-2. Effector cytokines make monocytes release inflammatory cytokines such as IL-1, IL-6, IFNγ and IL-10. These inflammatory cytokines can result in clinical manifestations of CRS mentioned above. Treatments including tocilizumab, corticosteriods, vasoactive drugs, mechanical ventilation and other supportive measures should be used for patients who developed severe CRS in time [34]. IL-6, as one inflammatory cytokine of CRS, leads to the clinical application of the IL-6 receptor inhibitor such as tocilizumab for the management of CRS [35]. Tocilizumab blocks membrane-bound as well as soluble IL-6 receptors. A retrospective analysis for patients with CRS after the treatment with CTL019 and KTE-C19 on prospective clinical trials showed that 69% of patients (31/45) in clinical trial of CTL019 had a response within 14 days of the first dose of tocilizumab. And 53% of patients (8/15) in the clinical trial of KTE-C19 received a response after treatment of tocilizumab. No tocilizumab-related adverse events were reported [36]. Corticosteriods are used in clinical experience due to the extensive effects on immune system and often used in tocilizumab-refractory cases. However, corticosteriods may influence the activation and proliferation of CAR-T cells [34]. Besides, many third-line agents such as siltuximab, cyclophosphamide and anakinra have been applied in cases of refractory CRS. Janus kinase–signal transducers and activators of transcription (JAK-STAT) pathway plays an important role on inducing biologic activity for many inflammatory cytokines and mediators. There was a phase II clinical trial of itacitinib, a JAK1 inhibitor, for the management of CRS induced by CAR-T cell therapy [37]. The clinical trial showed that itacitinib could reduce cytokines levels such as IL-6, IL-12, and IFN-γ in murine models of inflammation. Previous researches have demonstrated that host macrophages are the main source of IL-6 after CAR-T cells infusion [38]. Itacitinib can reduce the level of IL-6 released by macrophages and reduce cytokines production of CAR-T cells without affecting proliferation and anti-tumor activity of CAR-T cells [37]. Due to the specific role of macrophage in CRS, new therapies targeting macrophage-involved pathways such as GM-CSF and atrial natriuretic peptide (ANP) have great potential [39]. Besides, hemofiltration and plasma exchange can be used to control severe CRS in some patients which cannot be relieved by corticosteriods, tocilizumab and other treatments.

### ICANS

Neurotoxicity or ICANS is the second most common toxicity related to CAR-T cell therapy. ICANS typically occurs 4 to 5 days after CAR-T treatment, lasts 5 to 10 days and can range from disorientation and aphasia to potentially life-threatening brain edema. A variety of symptoms can be observed ranging from early symptoms such as word-finding difficulty, confusion, headache and impaired attention to more severe presentations including damaging motor skills, seizures, descending level of consciousness, coma, cerebral edema, and death. High tumor burden, high infused doses of CAR-T cells, high-intensity lymphodepletion and preexisting neurologic complications could be the risk factors for ICANS [32]. Serum biomarker analysis showed the associations of IL-6, IL-15, IL-2Rα, and other biomarkers with grade 3 or higher CRS and ICANS. However, CAR-T cells levels and some specific cytokines, including IL-2, GM-CSF, and ferritin, were associated only with grade 3 or higher ICANS, but not severe CRS [40, 41]. Besides, baseline level of blood platelet < 60 × 10^9^, mean corpuscular hemoglobin concentration (MCHC) > 33.2% and morphologic disease (> 5% blasts) can also serve as predictive biomarkers for severe neurotoxicity [32]. Patients with ICANS are difficult in having good response to treatment compared to patients with CRS. As a result, patients usually need more hospitalization time and supportive care [42]. The best treatment for ICANS is still unknown nowdays. Supportive therapy can manage patients with grade 1 or 2 ICANS. And patients with grade 3 or higher ICANS are usually treated with corticosteriods in addition to supportive therapy. Patients with grade 4 or more severe ICANS usually need therapy on the intensive care unit along with intubation and mechanical ventilation [43]. In patients with depressed level of consciousness, dexamethasone should be used to control the seizure of disease [44]. Tocilizumab, the IL-6 antagonist, is not recommended using for ICANS according to relevant studies because it has poor capacity of Blood-Brain-Barrier (BBB) penetration. Anakinra, an IL-1 receptor antagonist, have demonstrated great potential to prevent both CRS and ICANS in murine models [45]. Besides, lenzilumab, a neutralizing antibody against granulocyte-macrophage colony-stimulating factor (GM-CSF), also has shown great potency to relieve the severity of neurotoxicity in a phase II study [46]. Cerebral edema is the most serious complication of ICANS. In the clinical trial (JCAR015)for patients with ALL, 5 patients died of severe cerebral edema with blood-brain barrier disruption. Mannitol or hypertonic saline should be used to decrease cranial pressure of patients with cerebral edema [47]. Magnetic Resonance Imaging (MRI) and Electroencephalogram (EEG) abnormalities are common in patients with ICANS, which can help us detect and manage neurotoxicity after CAR-T cell therapy better [48].

### Other adverse events

Lymphodepletion can reduce the level of regulatory T cells and promote proliferation of CAR-T cells. However, lymphodepletion by fludarabine and cyclophosphamide may cause hematological abnormalities such as neutropenia, leukopenia, anemia, and thrombocytopenia. Off-target toxicity of CAR-T cell therapy is that CAR-T cells react with normal tissue without expression of targeting antigen. On-target off-tumor toxicity of CAR-T cell therapy is that CAR-T cells recognize and bind to targeted antigens expressed on normal cells, and the outcomes are more severe when occurred on treatment for solid tumors than hematological malignancies. In addition, on-target on-tumor toxicity is induced by rapid destruction of tumor cells. The release of contents in tumor cells can cause metabolic disorder and affect organ functions. On-target on-tumor toxicity, or tumor lysis syndrome, was reported in the CAR-T cell therapy for patients with ALL [12]. Tumor lysis syndrome of CAR-T cell therapy can result in life-threatening arrhythmias and renal failure when patients with high tumor burden. Therefore, lymphodepleting chemotherapy is important before administration of CAR-T cells in these patients. Infections are common after CAR-T cell therapy. The increased risk of infections are associated with the use of corticosteroids and tocilizumab. Monitoring and treatment of hypogammaglobulinemia, prevention and management of infections are important.

### Current problems of CAR-T cell therapy in B-NHL

Although CAR-T cell therapies have had a marked impact on the management of patients with B-cell malignancies including chemotherapy refractory aggressive B-NHL, initial CRs were observed in about 90% of patients with ALL in clinical trials while the CR rate of patients with B-NHL was inferior [49]. There are several reasons for the inferior responses. Lymphomas are solid tumors which have a physical barrier to prevent CAR-T cells to contact tumor cells. Besides, CAR-T cells have to overcome the immunosuppressive effects of regulatory T cells (Tregs), tumor-associated macrophages (TAMs), myeloid-derived suppressor cells (MDSCs) and inhibitory substances in the tumor microenvironment (TME). CAR-T cells could also lack chemokines to enter lymphoid tissues [50, 51]. Tregs play a vital role in maintaining immune tolerance to self-antigens but can also suppress antitumor immunity. One study found that antibody-mediated consumption of 4-1BB-expressing cells can decrease tumor growth on murine tumor models and 4–1 BB was highly selectively for human tumor Tregs [52]. The combination of CAR-T cells with corresponding antibodies may increase antitumor effects of CAR-T cell therapy. The TAMs are one of the components of non-tumor stromal cells in TME and play an important role in occurrence and progression of tumors including hematological malignancies [53, 54]. Combined with agents that target TAMs and developing CARs that target antigens expressed by TAMs can overcome the immunosuppression. The programmed cell death protein 1 (PD-1) and programmed death-ligand 1/2 (PD-L1/L2) play an important role in suppressing the antitumor immune response. The up-regulation of PD-1 is associated with T-cell exhaustion. Overexpression of PD-L1 in tumor cells has been confirmed to inhibit CAR-T cell function [55]. Blocking the pathway with immune checkpoint inhibitors can reverse T-cell exhaustion and increase the antitumor effects of CAR-T cell therapy [51, 56]. Furthermore, age of patient and T-cell quality may affect the function of CAR-T cell. A clinical trial analyzed differences in production features and phenotype of CD19 CAR-T cells with CD28 co-stimulatory domains between ALL and B-NHL patients. In the clinical trial, 100% of ALL patients and 94% of B-NHL patients received CAR-T cells at the target dose of 1 × 10^6 cells/kg. CAR-T cells in ALL patients expanded better than that in NHL patients. Besides, the CAR-T cells from ALL patients contained more naive T cells (TN) than CAR-T cells from B-NHL patients did. They also found that CAR-T cells of younger patients (< 20 years) demonstrated an increased fold expansion compared with older patients (≥20 years). The ORR was 84% (30/36) in ALL patients and 62% (32/52) in B-NHL patients [57]. TNcells (CD45RA + CCR7+) have long-term proliferation in vivo after administration, which can possibly enhance the clinical outcome of CAR-T cell therapy [58]. Analysis of the clinical trial demonstrated no significant differences in other phenotypes between ALL group and NHL group. The inferior response rate of CAR-T cell therapy for NHL patients may attribute to lower quality of CAR-T cells manufactured from patients with NHL. Further efforts is required to overcome the limitation. Manufacture failure and high cost of the therapy impact the clinical applications of CAR-T therapy. “Off-the-shelf” strategies and allogeneic CAR-T products could help us overcome these limitations [59, 60]. Besides, CAR-T cell-associated toxicities including CRS and ICANS have impeded the feasibility of CAR-T therapy and limited the number of patients who are eligible for this treatment. Although there are some solutions to deal with adverse events, new strategies which can prevent the occurrence of toxicities associated with CAR-T therapy are still needed. Modifying CAR structure could be one solution to decrease the incidence of adverse events. A phase I clinical trial found that a new anti-CD19 CAR-T cell (CD19-BBz(86)) with co-stimulatory 4-1BB and CD3ζ domains produced lower levels of cytokines. And no patients developed high-grade CRS or ICANS [61]. Utilizing humanized antibody fragments could decrease the immunogenicity of CAR and decrease the release of cytokines. For example, in the clinical trial of Hu19-CD828Z, designed with a scFv from a fully-human anti-CD19 monoclonal antibody and CD28 costimulatory domain, a lower incidence of high-grade ICANS than murine-derived CAR was observed [24].

### New developments of CAR-T cell therapy

Anti-CD19 CAR-T cell therapies have significantly improved efficacy in patients with refractory B-NHL. However, some patients relapsed after anti-CD19 CAR-T cell therapy. It is important to understand the mechanisms of relapse or non-response in patients with B-NHL after CAR-T cell therapy. There are two main mechanisms of relapse. The mechanisms of antigen positive relapse are associated with limited CAR-T cells persistence or B cell aplasia. The determinants of CAR-T cell persistence have yet to be fully determined. Internal quality of T cell and T cell phenotype could influence CAR-T cell persistence in vivo. And the mechanisms of antigen negative relapse are antigen down regulation and antigen loss. These limitations of current CAR-T cell therapy indicate the necessity of new-generation CAR-T cell therapy (Table 4).

**Table 4:** B cell lymphoma antigens targeted by CAR-T cells

CAR-T antigen	Clinical study	Disease
CD19	Safety and feasibility of anti-CD19 CAR T cells with fully human binding domains in patients with B-cell lymphoma	B-cell lymphoma; B-ALL
BAFF-R	Antitumor efficacy of BAFF-R targeting CAR T cells manufactured under clinic-ready conditions	B- ALL; B-NHL
CD20	Phase II trial of co-administration of CD19- and CD20-targeted chimeric antigen receptor T cells for relapsed and refractory diffuse large B cell lymphoma	R/R DLBCL
CD22	CD19/CD22 Dual-Targeted CAR-T Therapy Active in Relapsed/Refractory DLBCL	R/R DLBCL
CD79b	Targeting CD79b for Chimeric Antigen Receptor T-Cell Therapy of B-Cell Lymphomas	B-cell lymphoma
CD37	Preclinical development of CD37CAR T-cell therapy for treatment of B-cell lymphoma	R/R B-NHL
PD-1	CD19-Specific CAR-T Cells that Express a PD-1/CD28 Chimeric Switch-Receptor is Effective in Patients with PD-L1 Positive B-Cell Lymphoma	PD-L1 Positive B-Cell Lymphoma
Igκ	T lymphocytes redirected against the κ light chain of human immunoglobulin efficiently kill mature B lymphocyte-derived malignant cells	Low-grade NHL; B-CLL

Abbreviations: *CAR* chimeric antigen receptor, *B-ALL* B-cell acute lymphoblastic leukemia, *B-NHL* B-cell non-Hodgkin lymphoma, *R/R DLBCL* relapsed and refractory diffuse large B cell lymphoma, *PD-1* programmed cell death protein-1, *PD-L1* programmed death ligand 1, *B-CLL* B-cell chronic lymphocytic leukemia

### B-cell activating factor receptor (BAFF-R)-targeted CAR-T cell therapy

Although CAR-T cells have achieved great efficacy on B-cell malignancies, antigen loss and lack of therapeutic persistence could result in disease relapse, which indicate the need for novel target selection and for improving the efficacy and persistence of the CAR-T cells. B-cell activating factor (BAFF) is a member of the tumor necrosis factor (TNF) superfamily, and it can activate and promote proliferation of B lymphocytes [62]. BAFF-R is a pro-survival receptor expressed on most malignant B cells and plays an important role in the proliferation of malignant lymphoma cells. Although BAFF-R is widely expressed on B-cells, serum BAFF levels were elevated in B-NHL patients compared to BAFF levels in healthy donors. High BAFF levels indicated aggressive disease and poor response to therapy. The blockade of BAFF and BAFF-R can be a therapeutic strategy in B-NHL [63]. The BAFF-R-targeted CAR-T cells can eliminate human malignant B cells expressing BAFF-R. Besides, BAFF-R-targeted CAR-T cells can kill human lymphoma and ALL cells with CD19 antigen loss in murine models effectively [62].

### CD20-targeted CAR-T cell therapy

The majority of B-cell lymphomas express CD20 and the over-expression of CD20 could indicate highly progressive disease [64]. CD20 phosphorylation was reported to be higher in proliferating malignant B cells than normal B cells [65]. A phase I clinical trial studied the second generation anti-CD20 CAR-T cells in 7 patients with refractory and relapsed DLBCL. Six of seven patients could be evaluated after CAR-T therapy and five of whom experienced tumor regression. Four of six evaluable patients achieved PR. However, three of these four patients eventually relapsed [66].

### CD19/CD20-targeted CAR-T cell therapy

There was a phase II clinical trial of co-administration of anti-CD19 CAR-T cells and anti-CD20 CAR-T cells for patients with refractory and relaped DLBCL. Twenty-five patients were enrolled in this trial and 21 patients received CAR-T infusion. Seventeen patients reached OR with 11 patients in CR. And the toxicities were manageable [67]. The result indicated an accessible choice for patients with refractory or relapsed DLBCL. A phase I/IIa clinical trial was designed to evaluate the efficacy and safety of TanCAR7 T cells for patients with relapsed or refractory B-cell lymphoma. TanCAR7 T cells can target both CD19 and CD20 with co-stimulatory domain of 4-1BB. A total of 28 patients received the infusion of TanCAR7 cells. The first 7 patients were treated at a dose of 0.5–6 × 10^6 cells/kg, and 21 patients were treated at a dose of 1–8 × 10^6 cells/kg. The ORR was 79% and the CR rate was 71%. PFS rate was 64% at 12 months follow-up. CRS occurred in 50% of patients (14/28). Grade 3 or higher CRS were observed in 14% of patients. ICANS occurred in 6 patients. And no patients developed grade 3 or higher ICANS [68].

### CD22-targeted CAR-T cell therapy

CD22, a member of sialic acid-binding immunoglobulin-like lectin (Siglec) family, is an inhibitory co-receptor of B-cell receptor that is exclusively expressed on B-cells. CD22 is highly expressed on B-cell lymphomas and leukemias so it has become an therapeutic target of cell therapy [69]. A phase I dose escalation study evaluated the efficacy and safety of CD22-targeted CAR-T cell therapy in patients with large B-cell lymphoma who relapsed following anti-CD19 CAR-T therapy. Three patients whose disease progressed after multiple treatments including previous anti-CD19 CAR-T therapy were enrolled. All patients received CR and no severe adverse events occurred after infusion of CD22 CAR-T cells. The study showed that CD22 could serve as a potential target of CAR-T cell therapy in large B-cell lymphoma [70].

### CD19/CD22 dual-targeted CAR-T cell therapy

Clinical trials of CD19/CD22 bispecific CAR-T cell therapy have manifested encouraging efficacy in B-cell malignancies. One patient with B-cell acute lymphoblastic leukemia (B-ALL) has maintained minimal residual disease (MRD)-negative remission for over 14 months after CD19/CD22 bispecific CAR-T cell therapy [71]. Besides, there is an ongoing phase I/II research studies the efficacy and safety of AUTO3, a bispecific CAR-T targeting both CD19 and CD22, in patients with relapsed/refractory DLBCL. Twenty-three patients were recruited and 16 patients received AUTO3. Sixty-nine percent of patients received OR and 56% of patients reached CR among 16 patients with DLBCL. There was no high grade adverse events related to AUTO3 treatment. Besides, there was another clinical trial that aimed at the efficacy and adverse events of CD19/CD22 CAR-T cell therapy for aggressive B-cell lymphoma involving gastrointestinal tract. Ten patients got OR and 7 patients achieved CR among all 14 recruited individuals with B-cell lymphoma involving gastrointestinal tract. CRS and gastrointestinal tract adverse events were manageable [72]. However, anti-CD22 agents cannot differentiate normal B cells from malignant B cells, resulting in side effects such as hemocytopenia. More strategies need to be developed to overcome the limitation of CD22-targeted CAR-T cell therapy.

### Trispecific CD19-CD20-CD22-targeted duoCAR-T

A preclinical research engineered trispecific duoCAR-T cells that target CD19, CD20, and CD22. The duoCAR consisted of a CAR that can target CD19 and CD20, linked by the P2A self-cleaving peptide to a second CAR targeting CD22. In the murine models bearing a mixture of B-cell lymphoma lines composed of CD19 negative, CD20 negative, and CD22 negative variants, the trispecific duoCAR-T cells could efficiently kill tumor cells. In contrast, monoCAR-T cells such as CD19-targeted CAR-T cells failed to prevent tumor progression. The research demonstrated that multispecific CAR-T cells can be a promising method to prevent antigen down regulation or antigen loss relapse [73].

### CD79b-targeted CAR-T cell therapy

B-cell receptor (BCR) is important for persistence and development of mature B cells and plays an essential role on tumorigenesis of B-cell lymphoma. CD79b, a signaling part of B-cell receptor, is a B-cell-restricted surface antigen expressed on both mature B cells and B-cell-derived malignancies such as B-NHL. One study detected the expression of CD79b on patients with different types of B-cell malignancies including DLBCL, Burkitt lymphoma, FL, and evaluated the efficacy of anti-CD79b CAR-T cells in vitro and in vivo. They found that the expression of CD79b on tumor cells of patients with different types of B-cell malignancies was wide. The CD79b mean fluorescence intensities (MFIs) of malignant lymphoma cells were similar to or higher than the MFI of normal B cells. Anti-CD79b CAR-T cells showed remarkable efficacy on tumors cells [74].

### CD37-targeted CAR-T cell therapy

CD37 is a member of tetraspanin superfamily mainly expressed on mature B cells including normal and malignancy B cells. The expression of CD37 in normal tissues is restricted to lymphoid organs such as spleen, lymph nodes and bone marrow [75]. High expression of CD37 has been detected across multiple types of B-NHL, which makes CD37 a potential target of immunotherapy for B-NHL patients [76]. The function of CD37 is not fully understood, but researches indicated that CD37 was involved in immune regulation and tumor supression [77]. CD37 is one of the potential targets for immunotherapy of B-NHL and some studies are ongoing, for example, 212Pb-NNV003, IMGN529, AGS67E, BI 836826 and anti-CD37 CAR-T. A preclinical study confirmed the high expression of CD37 on tumor cells of DLBCL, FL, MCL, MZL and CLL, which demonstrated the remarkable efficacy of anti-CD37 CAR-T to both CD19 positive B-cell lymphoma cell line and CD19 negative cell line in vitro. Besides, the CAR-T product reduced the growth of tumor cells in murine models and prolonged survival of mice, regardless of CD19 expression [76].

### PD-1 and CAR-T

**Fig. 3:**
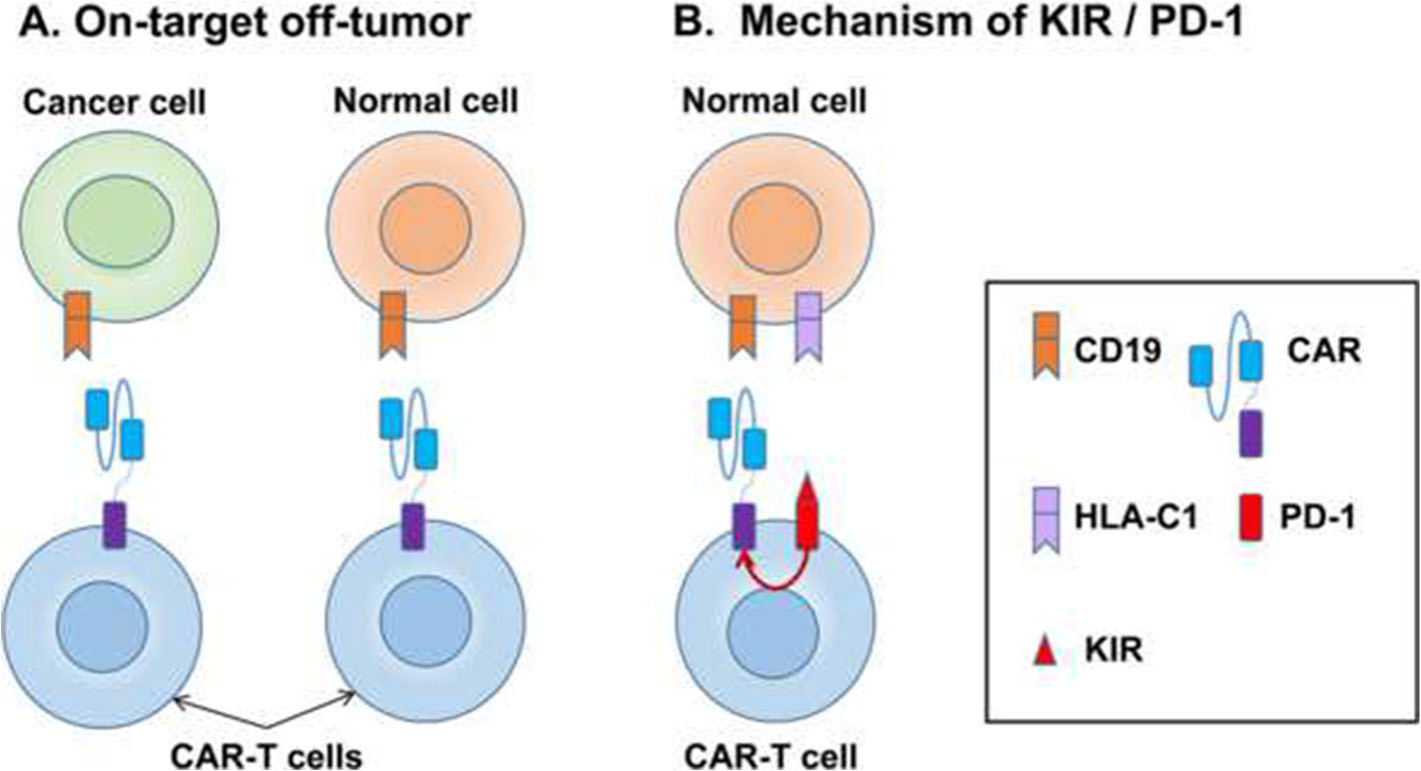
The mechanism of on-target off-tumor and killer inhibitory receptor (KIR) / PD-1-based inhibitory anti-CD19 CAR-T. **a** CAR-T cells can recognize and bind to targeted antigens (CD19) expressed on normal cells and cause on-target off-tumor toxicity; **b** The activation of CAR-T cells can be inhibited by PD-1 signal when KIR on CAR-T cells are engaged to HLA-C1 on normal cells, which can avoid the destruction of normal cells and on-target off-tumor toxicity of CAR-T cell therapy

PD-1 and PD-L1/L2 have been proved the important role on tumor immune escape, tumor progression and tumor survivals in various malignancies. And many experiments showed the highly expressed PD-L1 on lymphoma cells and up-regulated level of PD-1 in tumor-infiltrating lymphocytes, which indicates that PD-1/PD-L1 could be a therapeutic target for lymphoma. PD-1 is mainly expressed on activated T cells, B cells, and natural killer cells. PD-L1 or PD-L2 are mainly expressed on varieties of immune cells and tumor cells. High frequency of PD-L1 positivity has been detected in many types of B-cell lymphomas. The expression of PD-L1 was detected in 80 of 260 patients with DLBCL in one study [78]. There was a multicenter study of anti-CD19 CAR-T cells expressing PD-1/CD28 chimeric switch-receptor in 17 patients with large B-cell lymphoma including 15 PD-L1+ cases. Ten patients received OR and 7 of them received CR, while no severe ICANS or CRS occurred. This study showed the encouraging efficacy and safety of the anti-CD19 CAR-T cells expressing PD-1/CD28 chimeric switch-receptor. Besides, combination of CAR-T cell therapy and PD-1 blockade such as nivolumab and pembrolizumab have been applied to enhance anti-tumor efficacy in murine models and clinical trials. Eleven patients with relapsed/refractory lymphoma received CAR-T cells infusion at a dose of 5–11 × 10^6 cells/kg. All 11 patients were infused with nivolumab at a dose of 3 mg/kg after 3 days of CD19 CAR-T cells infusion. Nine patients (81.8%) achieved OR with 5 patients (45.5%) in CR. CRS occurred in 9 patients and all CRS were grade 1 or 2. Besides, no patients developed ICANS in the study. Other adverse events such as cytopenia and febrile syndrome were manageable [79, 80]. Furthermore, PD-1 not only can enhance the anti-tumor effect of CAR-T, but also can avoid B-cell aplasia via killer inhibitory receptor (KIR)/PD-1-based inhibitory anti-CD19 CAR-T. When KIR is engaged to human leukocyte antigen C1 (HLA-C1) on normal B-cells, it will deliver an inhibitory signaling via the intracellular PD-1 domain to avoid the destruction of normal B-cells caused by CAR-T cells (Fig. 3) [81].

### Multi-antigen-targeted CAR-T

Relevant studies have shown that antigen escape and on-target, off-tumor toxicities of CAR-T limit the application greatly [82]. Multi-antigen-targeted CAR-T, a new developing direction of CAR-T, is potential in overcoming antigen escape and on-target, off-tumor toxicities of previous CAR-T therapies. Multi-antigen-targeted CAR-T therapies mainly include pooled CAR-T cells, dual CAR-T cells, tandem CAR-T cells and trivalent CAR-T cells. Pooled CAR-T cells are a mixture of two CAR-T cell lines that target different antigens. Dual CAR-T is that two different chimeric antigen receptors are expressed in a single engineered T-cell. Tandem CAR-T is that two antigen-binding domains are connected in tandem to a single CAR. And trivalent CAR-T is that three distinct CARs are expressed in a single T cell. Multi-antigen-targeted CAR-T using the “OR” logic gate can effectively mitigate antigen escape and enhance anti-tumor efficacy while the “AND” and “NOT” logic-gated CAR-T cells can modulate the relationship between anti-tumor effect and on-target, off-tumor toxicities [83].

### Armored CAR-T cells and fourth-generation CAR-T

The armored CAR-T cell consists of two co-stimulatory domains, for example, CD28/4-1BB/CD3*ζ* or CD28/OX-40/CD3*ζ*, while second-generation CAR-T cell, the most widely used CAR-T in clinical trials, consists of one co-stimulatory domain. In comparison to conventional CAR-T, the armored CAR-T cells can achieve stronger levels of proliferation, persistence and IL-2 production. The armored CAR-T cells have superior activity and persistence in murine lymphoma models. However, they have not been widely applied in clinical trials of human [84, 85]. Fourth-generation CAR-T is called T cell redirected for antigen-unrestricted cytokine-initiated killing (TRUCKs), which can produce and release transduced cytokines such as IL-12, IL-15 or IL-18 to enhance the activity of CAR-T cells, change the tumor environment and achieve self-activation by autocrine pathways [86, 87].

### Allogeneic CAR-T cell therapy and universal CARs

Autologous CAR-T therapies have achieved great clinical progress in B-NHL. However, some patients may fail to receive CAR-T cells infusion as a result of manufacturing failures or disease progression during the manufacturing process. Besides, many patients cannot afford the high cost of the therapy. These limitations impact the clinical applications of autologous CAR-T therapy [88]. Allogeneic CAR-T cell therapy uses T-cells collected from healthy donors, which could overcome the disfunction of T-cells from patients. And the cost of CAR-T therapy can be decreased due to the industrial production of donor-drived T-cells. However, there are still some problems exist in allogeneic CAR-T cell therapy. One of the predominant problems are the risk of graft-versus-host disease (GVHD). Besides, the allogeneic T-cells could be eliminated by the immune systems of recipients, which could impact the antitumor efficacy of CAR-T cells. Eliminating the expression of T-cell receptor (TCR) and CD52 by gene editing technologies or other feasible approaches, for example, adding a suicide gene such as inducible caspase 9 (iC9) to the allogeneic CAR-T cells, could manage these adverse effects [60, 89]. Besides, the conventional design of chimeric antigen receptor and manufacturing process of CAR-T cells lead to the expensive cost of this therapy, which limits the clinical uses too. New chimeric antigen receptors can be designed with a modular method. The antigen recognition domain of CAR-T cell is separated from the signaling domain of CAR-T cell so that the antigen can be changed without the need for redesigning the CAR-T cells. This new CAR can serve as a universal CAR (UniCAR). The UniCAR-T cells remain inactive until bispecific switch molecules correlate the CAR-T cells with tumor cells expressing targeting antigens, and the degree of response can be regulated by adjusting the concentration of these molecules to balance the efficacy and adverse effects of CAR-T cell therapy [90].

## Conclusion

In summary, for those patients with relapsed or refractory B-NHL who had unsatisfactory prognosis treated by chemotherapeutic regimens and other new therapeutic agents, CAR-T cell therapy, especially anti-CD19 CAR-T cell therapy, has demonstrated great efficacy in plenty of clinical or preclinical trials. And four CAR-T cell products have been approved by FDA and/or EMA. Outcomes in adults with B-NHL are variable with CR rates ranging from 52 to 82%. The construct of CAR-T product, bridging therapy, inflammation and tumor burden can affect the outcomes of this therapy. However, there exist some problems in spite of the great efficacy. CRS, ICANS and other adverse events such as neutropenia and off-target toxicity were observed. The occurrence rates of CRS range from 42 to 93% and the rates of grade 3 or higher CRS range from 2 to 22%. Low-grade adverse events are manageable while severe side effects should be treated by tocilizumab, corticosteriods, vasoactive drugs, mechanical ventilation and other supportive measures. Hemofiltration and plasma exchange can be alternative therapeutic methods for severe CRS. Besides, the outcomes of patients with B-NHL treated by CAR-T cell therapy were inferior to patients with ALL. Physical barrier of B-NHL, tumor microenvironment and inherent quality of T cells may explain the inferior response rate. Furthermore, parts of patients relapsed after anti-CD19 CAR-T cell therapy, which were due to limited of CAR-T cells persistence, CD19 antigen loss or antigen down-regulation of tumor cells. To overcome the limitation of anti-CD19 CAR-T, developments of new generation of the therapy are ongoing. For example, CD20-targeted CAR-T, CD19/CD22 dual-targeted CAR-T, CD79b-targeted CAR-T, CD37-targeted CAR-T and CAR-T expressing PD-1/CD28 chimeric switch-receptor have demonstrated encouraging effects. Third-generation CAR-T and armored CAR-T can achieve stronger levels of proliferation, persistence and IL-2 production than previous CAR-T cell in vitro or vivo. Besides, the allogeneic CAR-T cell therapy could make the therapy available to more patients. Furthermore, researchers should try their best to improve the safety and efficacy of CAR-T to make patients benefit from this powerful weapon against B-cell malignancies.

## Data Availability

Not applicable.

## Abbreviations

CAR-T: Chimeric antigen receptor T-cell
B-NHL: B-cell non-Hodgkin lymphoma
ScFv: Single chain variable fragments
FDA: The United States Food and Drug Administration
EMA: European Medicines Agency
MCL: Mantle cell lymphoma
OR: Objective response
CR: Complete response
ORR: Objective response rate
PFS: Progression-free survival
DLBCL: Diffuse large B-cell lymphoma
FL: Follicular lymphoma
ASCT: Autologous hematopoietic stem-cell transplantation
CRS: Cytokine release syndrome
ICANS: Immune effector cell-associated neurotoxicity syndrome
CRP: C-reactive protein
TNFα: Tumor necrosis factor alpha
IFNγ: Interferon gamma
GM-CSF: Granulocyte-macrophage Colony Stimulating Factor
ANP: Atrial natriuretic peptide
JAK-STAT: Janus kinase–signal transducers and activators of transcription
BAFF-R: B-cell activating factor receptor
Siglec: Sialic acid-binding immunoglobulin-like lectin
PD-1: Programmed cell death protein-1
PD-L1/L2: Programmed cell death protein ligand 1/2
HLA: Human leukocyte antigen
KIR: Killer inhibitory receptors
TRUCKs: T cell redirected for antigen-unrestricted cytokine-initiated killing
GVHD: Graft-versus-host disease
iC9: Inducible caspase 9
Tregs: Regulatory T cells
TME: Tumor microenvironment
TAMs: Tumor-associated macrophages
MDSCs: Myeloid-derived suppressor cells
TN: Naive T cells
TCM: Central memory T cells
TEM: Effector memory T cells
PFS: Progression-free survival
ALL: Acute lymphoblastic leukemia

## References

[1] Yang H, Green MR. Epigenetic programing of B-cell lymphoma by BCL6 and its genetic deregulation. Front Cell Dev Biol. 2019;7. 10.3389/fcell.2019.00272. 10.3389/fcell.2019.00272 PMC6853842 31788471

[2] Roschewski M, Staudt LM, Wilson WH (2014). Diffuse large B-cell lymphoma-treatment approaches in the molecular era. Nat Rev Clin Oncol.

[3] Linschoten M, Kamphuis J, van Rhenen A, Bosman L, Cramer M, Doevendans P, Teske A, Asselbergs F (2020). Cardiovascular adverse events in patients with non-Hodgkin lymphoma treated with first-line cyclophosphamide, doxorubicin, vincristine, and prednisone (CHOP) or CHOP with rituximab (R-CHOP): a systematic review and meta-analysis. Lancet Haematol.

[4] Mondello P, Mian M (2019). Frontline treatment of diffuse large B-cell lymphoma: beyond R-CHOP. Hematol Oncol.

[5] Brudno JN, Kochenderfer JN (2018). Chimeric antigen receptor T-cell therapies for lymphoma. Nat Rev Clin Oncol.

[6] van Dorsten R, Lambson B, Wibmer C, Weinberg M, Moore P, Morris L. Neutralization Breadth and Potency of Single-Chain Variable Fragments Derived from Broadly Neutralizing Antibodies Targeting Multiple Epitopes on the HIV-1 Envelope. J Virol. 2020;94(2):e01533–19. 10.1128/JVI.01533-19 PMC6955269 31619559

[7] Johnson P, Abramson J (2020). Patient selection for chimeric antigen receptor (CAR) T-cell therapy for aggressive B-cell non-Hodgkin lymphomas. Leuk Lymphoma.

[8] Bachanova V, Perales M, Abramson J (2020). Modern management of relapsed and refractory aggressive B-cell lymphoma: a perspective on the current treatment landscape and patient selection for CAR T-cell therapy. Blood Rev.

[9] Schuster SJ, Svoboda J, Chong EA, Nasta SD, Mato AR, Anak O, Brogdon JL, Pruteanu-Malinici I, Bhoj V, Landsburg D, Wasik M, Levine BL, Lacey SF, Melenhorst JJ, Porter DL, June CH (2017). Chimeric antigen receptor T cells in refractory B-cell lymphomas. N Engl J Med.

[10] Gofshteyn JS, Shaw PA, Teachey DT, Grupp SA, Maude S, Banwell B, Chen F, Lacey SF, Melenhorst JJ, Edmonson MJ (2018). Neurotoxicity after CTL019 in a pediatric and young adult cohort. Ann Neurol.

[11] Lee DW, Kochenderfer JN, Stetler-Stevenson M, Cui YK, Delbrook C, Feldman SA, Fry TJ, Orentas R, Sabatino M, Shah NN, Steinberg SM, Stroncek D, Tschernia N, Yuan C, Zhang H, Zhang L, Rosenberg SA, Wayne AS, Mackall CL (2015). T cells expressing CD19 chimeric antigen receptors for acute lymphoblastic leukaemia in children and young adults: a phase 1 dose-escalation trial. Lancet.

[12] Maude SL, Frey N, Shaw PA, Aplenc R, Barrett DM, Bunin NJ, Chew A, Gonzalez VE, Zheng Z, Lacey SF, Mahnke YD, Melenhorst JJ, Rheingold SR, Shen A, Teachey DT, Levine BL, June CH, Porter DL, Grupp SA (2014). Chimeric antigen receptor T cells for sustained remissions in leukemia. N Engl J Med.

[13] Danylesko I, Chowers G, Shouval R, Besser M, Jacoby E, Shimoni A, Nagler A, Avigdor A (2020). Treatment with anti CD19 chimeric antigen receptor T cells after antibody-based immunotherapy in adults with acute lymphoblastic leukemia. Curr Res Transl Med.

[14] Klener P. Advances in Molecular Biology and Targeted Therapy of Mantle Cell Lymphoma. Int J Mol Sci. 2019;20(18):4417. 10.3390/ijms20184417 PMC6770169 31500350

[15] Gerson JN, Barta SK (2019). Mantle cell lymphoma: which patients should we transplant?. Curr Hematol Malig Rep.

[16] Arora PC, Portell CA (2018). Novel therapies for relapsed/refractory mantle cell lymphoma. Best Pract Res Clin Haematol.

[17] Wang M, Munoz J, Goy A, Locke FL, Jacobson CA, Hill BT, Timmerman JM, Holmes H, Jaglowski S, Flinn IW, McSweeney PA, Miklos DB, Pagel JM, Kersten MJ, Milpied N, Fung H, Topp MS, Houot R, Beitinjaneh A, Peng W, Zheng L, Rossi JM, Jain RK, Rao AV, Reagan PM (2020). KTE-X19 CAR T-cell therapy in relapsed or refractory mantle-cell lymphoma. N Engl J Med.

[18] Lossos IS (2005). Molecular pathogenesis of diffuse large B-cell lymphoma. J Clin Oncol.

[19] Locke FL, Neelapu SS, Bartlett NL, Siddiqi T, Chavez JC, Hosing CM, Ghobadi A, Budde LE, Bot A, Rossi JM, Jiang Y, Xue AX, Elias M, Aycock J, Wiezorek J, Go WY (2017). Phase 1 results of ZUMA-1: a multicenter study of KTE-C19 anti-CD19 CAR T cell therapy in refractory aggressive lymphoma. Mol Ther.

[20] Neelapu S, Locke F, Bartlett N, Lekakis L, Miklos D, Jacobson C, Braunschweig I, Oluwole O, Siddiqi T, Lin Y (2017). Axicabtagene Ciloleucel CAR T-cell therapy in refractory large B-cell lymphoma. N Engl J Med.

[21] Locke FL, Ghobadi A, Jacobson CA, Miklos DB, Lekakis LJ, Oluwole OO, Lin Y, Braunschweig I, Hill BT, Timmerman JM, Deol A, Reagan PM, Stiff P, Flinn IW, Farooq U, Goy A, McSweeney PA, Munoz J, Siddiqi T, Chavez JC, Herrera AF, Bartlett NL, Wiezorek JS, Navale L, Xue A, Jiang Y, Bot A, Rossi JM, Kim JJ, Go WY, Neelapu SS (2019). Long-term safety and activity of axicabtagene ciloleucel in refractory large B-cell lymphoma (ZUMA-1): a single-arm, multicentre, phase 1–2 trial. Lancet Oncol.

[22] Kochenderfer JN, Somerville RPT, Lu T, Shi V, Bot A, Rossi J, Xue A, Goff SL, Yang JC, Sherry RM, Klebanoff CA, Kammula US, Sherman M, Perez A, Yuan CM, Feldman T, Friedberg JW, Roschewski MJ, Feldman SA, McIntyre L, Toomey MA, Rosenberg SA (2017). Lymphoma remissions caused by anti-CD19 chimeric antigen receptor T cells are associated with high serum Interleukin-15 levels. J Clin Oncol.

[23] Alabanza L, Pegues M, Geldres C, Shi V, Wiltzius JJW, Sievers SA, Yang S, Kochenderfer JN (2017). Function of novel anti-CD19 chimeric antigen receptors with human variable regions is affected by hinge and transmembrane domains. Mol Ther.

[24] Brudno JN, Lam N, Vanasse D, Shen Y-W, Rose JJ, Rossi J, Xue A, Bot A, Scholler N, Mikkilineni L (2020). Safety and feasibility of anti-CD19 CAR T cells with fully human binding domains in patients with B-cell lymphoma. Nat Med.

[25] Schuster SJ, Bishop MR, Tam CS, Waller EK, Borchmann P, McGuirk JP, Jäger U, Jaglowski S, Andreadis C, Westin JR (2019). Tisagenlecleucel in adult relapsed or refractory diffuse large B-cell lymphoma. N Engl J Med.

[26] Abramson J, Palomba M, Gordon L, Lunning M, Wang M, Arnason J, Mehta A, Purev E, Maloney D, Andreadis C (2020). Lisocabtagene maraleucel for patients with relapsed or refractory large B-cell lymphomas (TRANSCEND NHL 001): a multicentre seamless design study. Lancet (London, England).

[27] Pinnix C, Gunther J, Dabaja B, Strati P, Fang P, Hawkins M, Adkins S, Westin J, Ahmed S, Fayad L (2020). Bridging therapy prior to axicabtagene ciloleucel for relapsed/refractory large B-cell lymphoma. Blood Adv.

[28] Locke FL, Rossi JM, Neelapu SS, Jacobson CA, Miklos DB, Ghobadi A, Oluwole OO, Reagan PM, Lekakis LJ, Lin Y, Sherman M, Better M, Go WY, Wiezorek JS, Xue A, Bot A (2020). Tumor burden, inflammation, and product attributes determine outcomes of axicabtagene ciloleucel in large B-cell lymphoma. Blood Adv.

[29] Cao J-X, Wang H, Gao W-J, You J, Wu L-H, Wang Z-X (2020). The incidence of cytokine release syndrome and neurotoxicity of CD19 chimeric antigen receptor–T cell therapy in the patient with acute lymphoblastic leukemia and lymphoma. Cytotherapy.

[30] Neelapu SS, Tummala S, Kebriaei P, Wierda W, Gutierrez C, Locke FL, Komanduri KV, Lin Y, Jain N, Daver N, Westin J, Gulbis AM, Loghin ME, de Groot JF, Adkins S, Davis SE, Rezvani K, Hwu P, Shpall EJ (2017). Chimeric antigen receptor T-cell therapy — assessment and management of toxicities. Nat Rev Clin Oncol.

[31] Liu D, Zhao J (2018). Cytokine release syndrome: grading, modeling, and new therapy. J Hematol Oncol.

[32] Wang Z, Han W (2018). Biomarkers of cytokine release syndrome and neurotoxicity related to CAR-T cell therapy. Biomark Res.

[33] Sievers S, Watson G, Johncy S, Adkins S (2020). Recognizing and grading CAR T-cell toxicities: an advanced practitioner perspective. Front Oncol.

[34] Chen H, Wang F, Zhang P, Zhang Y, Chen Y, Fan X, Cao X, Liu J, Yang Y, Wang B, Lei B, Gu L, Bai J, Wei L, Zhang R, Zhuang Q, Zhang W, Zhao W, He A (2019). Management of cytokine release syndrome related to CAR-T cell therapy. Front Med.

[35] Kotch C, Barrett D, Teachey DT (2019). Tocilizumab for the treatment of chimeric antigen receptor T cell-induced cytokine release syndrome. Expert Rev Clin Immunol.

[36] Le RQ, Li L, Yuan W, Shord SS, Nie L, Habtemariam BA, Przepiorka D, Farrell AT, Pazdur R (2018). FDA approval summary: tocilizumab for treatment of chimeric antigen receptor T cell-induced severe or life-threatening cytokine release syndrome. Oncologist.

[37] Huarte E, O'Conner RS, Peel MT, Nunez-Cruz S, Leferovich J, Juvekar A, et al. Itacitinib (INCB039110), a JAK1 inhibitor, Reduces Cytokines Associated with Cytokine Release Syndrome Induced by CAR T-Cell Therapy. Clin Cancer Res. 2020;26(23):6299–309. 10.1158/1078-0432.CCR-20-1739 PMC7895329 32998963

[38] Norelli M, Camisa B, Barbiera G, Falcone L, Purevdorj A, Genua M, Sanvito F, Ponzoni M, Doglioni C, Cristofori P, Traversari C, Bordignon C, Ciceri F, Ostuni R, Bonini C, Casucci M, Bondanza A (2018). Monocyte-derived IL-1 and IL-6 are differentially required for cytokine-release syndrome and neurotoxicity due to CAR T cells. Nat Med.

[39] Hao Z, Li R, Meng L, Han Z, Hong Z (2020). Macrophage, the potential key mediator in CAR-T related CRS. Exp Hematol Oncol.

[40] Karschnia P, Jordan J, Forst D, Arrillaga-Romany I, Batchelor T, Baehring J, Clement N, Gonzalez Castro L, Herlopian A, Maus M (2019). Clinical presentation, management, and biomarkers of neurotoxicity after adoptive immunotherapy with CAR T cells. Blood.

[41] Du M, Hari P, Hu Y, Mei H (2020). Biomarkers in individualized management of chimeric antigen receptor T cell therapy. Biomark Res.

[42] Rubin D, Danish H, Ali A, Li K, LaRose S, Monk A, Cote D, Spendley L, Kim A, Robertson M (2019). Neurological toxicities associated with chimeric antigen receptor T-cell therapy. Brain J Neurol.

[43] Neelapu S (2019). Managing the toxicities of CAR T-cell therapy. Hematol Oncol.

[44] Azoulay E, Darmon M, Valade S (2020). Acute life-threatening toxicity from CAR T-cell therapy. Intensive Care Med.

[45] Strati P, Ahmed S, Kebriaei P, Nastoupil L, Claussen C, Watson G, Horowitz S, Brown A, Do B, Rodriguez M (2020). Clinical efficacy of anakinra to mitigate CAR T-cell therapy-associated toxicity in large B-cell lymphoma. Blood Adv.

[46] Sterner R, Sakemura R, Cox M, Yang N, Khadka R, Forsman C, Hansen M, Jin F, Ayasoufi K, Hefazi M (2019). GM-CSF inhibition reduces cytokine release syndrome and neuroinflammation but enhances CAR-T cell function in xenografts. Blood.

[47] Rice J, Nagle S, Randall J, Hinson H (2019). Chimeric antigen receptor T cell-related neurotoxicity: mechanisms, clinical presentation, and approach to treatment. Curr Treat Options Neurol.

[48] Strati P, Nastoupil L, Westin J, Fayad L, Ahmed S, Fowler N, Hagemeister F, Lee H, Iyer S, Nair R (2020). Clinical and radiologic correlates of neurotoxicity after axicabtagene ciloleucel in large B-cell lymphoma. Blood Adv.

[49] Mohty M, Gautier J, Malard F, Aljurf M, Bazarbachi A, Chabannon C, Kharfan-Dabaja M, Savani B, Huang H, Kenderian S (2019). CD19 chimeric antigen receptor-T cells in B-cell leukemia and lymphoma: current status and perspectives. Leukemia.

[50] Enblad G, Karlsson H, Loskog A (2015). CAR T-cell therapy: the role of physical barriers and immunosuppression in lymphoma. Hum Gene Ther.

[51] Rodriguez-Garcia A, Palazon A, Noguera-Ortega E, Powell D, Guedan S (2020). CAR-T cells hit the tumor microenvironment: strategies to overcome tumor escape. Front Immunol.

[52] Freeman Z, Nirschl T, Hovelson D, Johnston R, Engelhardt J, Selby M, Kochel C, Lan R, Zhai J, Ghasemzadeh A (2020). A conserved intratumoral regulatory T cell signature identifies 4-1BB as a pan-cancer target. J Clin Invest.

[53] Ge Z, Ding S (2020). The crosstalk between tumor-associated macrophages (TAMs) and tumor cells and the corresponding targeted therapy. Front Oncol.

[54] Komohara Y, Niino D, Ohnishi K, Ohshima K, Takeya M (2015). Role of tumor-associated macrophages in hematological malignancies. Pathol Int.

[55] Qin L, Zhao R, Chen D, Wei X, Wu Q, Long Y, Jiang Z, Li Y, Wu H, Zhang X, Wu Y, Cui S, Wei W, Yao H, Liu Z, Cao S, Yao Y, Zhang Z, Li P (2020). Chimeric antigen receptor T cells targeting PD-L1 suppress tumor growth. Biomark Res.

[56] Boussiotis V (2016). Molecular and biochemical aspects of the PD-1 checkpoint pathway. N Engl J Med.

[57] Itzhaki O, Jacoby E, Nissani A, Levi M, Nagler A, Kubi A, et al. Head-to-head comparison of in-house produced CD19 CAR-T cell in ALL and NHL patients. J Immunother Cancer. 2020;8(1):e000148. 10.1136/jitc-2019-000148 PMC7061891 32152221

[58] Stock S, Schmitt M, Sellner L. Optimizing Manufacturing Protocols of Chimeric Antigen Receptor T Cells for Improved Anticancer Immunotherapy. Int J Mol Sci. 2019;20(24):6223. 10.3390/ijms20246223 PMC6940894 31835562

[59] Perez C, Gruber I, Arber C (2020). Off-the-shelf allogeneic T cell therapies for Cancer: opportunities and challenges using naturally occurring "universal" donor T cells. Front Immunol.

[60] Depil S, Duchateau P, Grupp S, Mufti G, Poirot L (2020). Off-the-shelf' allogeneic CAR T cells: development and challenges. Nat Rev Drug Discov.

[61] Ying Z, Huang XF, Xiang X, Liu Y, Kang X, Song Y, Guo X, Liu H, Ding N, Zhang T, Duan P, Lin Y, Zheng W, Wang X, Lin N, Tu M, Xie Y, Zhang C, Liu W, Deng L, Gao S, Ping L, Wang X, Zhou N, Zhang J, Wang Y, Lin S, Mamuti M, Yu X, Fang L, Wang S, Song H, Wang G, Jones L, Zhu J, Chen SY (2019). A safe and potent anti-CD19 CAR T cell therapy. Nat Med.

[62] Qin H, Dong Z, Wang X, Cheng W, Wen F, Xue W, et al. CAR T cells targeting BAFF-R can overcome CD19 antigen loss in B cell malignancies. Sci Transl Med. 2019;11(511):eaaw9414. 10.1126/scitranslmed.aaw9414 PMC7015136 31554741

[63] Yang S, Li J, Xu W (2014). Role of BAFF/BAFF-R axis in B-cell non-Hodgkin lymphoma. Crit Rev Oncol Hematol.

[64] Zettlitz K, Tavaré R, Tsai W, Yamada R, Ha N, Collins J, van Dam R, Timmerman J, Wu A (2019). F-labeled anti-human CD20 cys-diabody for same-day immunoPET in a model of aggressive B cell lymphoma in human CD20 transgenic mice. Eur J Nucl Med Mol Imaging.

[65] Pavlasova G, Mraz M (2020). The regulation and function of CD20: an "enigma" of B-cell biology and targeted therapy. Haematologica.

[66] Wang Y, Zhang W, Han Q, Liu Y, Dai H, Guo Y, Bo J, Fan H, Zhang Y, Zhang Y (2014). Effective response and delayed toxicities of refractory advanced diffuse large B-cell lymphoma treated by CD20-directed chimeric antigen receptor-modified T cells. Clin Immunol (Orlando, Fla).

[67] Sang W, Shi M, Yang J, Cao J, Xu L, Yan D, Yao M, Liu H, Li W, Zhang B, Sun K, Song X, Sun C, Jiao J, Qin Y, Sang T, Ma Y, Wu M, Gao X, Cheng H, Yan Z, Li D, Sun H, Zhu F, Wang Y, Zeng L, Li Z, Zheng J, Xu K (2020). Phase II trial of co-administration of CD19- and CD20-targeted chimeric antigen receptor T cells for relapsed and refractory diffuse large B cell lymphoma. Cancer Med.

[68] Tong C, Zhang Y, Liu Y, Ji X, Zhang W, Guo Y, Han X, Ti D, Dai H, Wang C, Yang Q, Liu W, Wang Y, Wu Z, Han W (2020). Optimized tandem CD19/CD20 CAR-engineered T cells in refractory/relapsed B-cell lymphoma. Blood.

[69] Peng W, Paulson J (2017). CD22 ligands on a natural N-glycan scaffold efficiently deliver toxins to B-lymphoma cells. J Am Chem Soc.

[70] Baird JH, Frank MJ, Craig J, Patel S, Spiegel JY, Sahaf B, Oak JS, Younes SF, Ozawa MG, Yang E, Natkunam Y, Tamaresis J, Ehlinger Z, Reynolds WD, Arai S, Johnston L, Lowsky R, Meyer E, Negrin RS, Rezvani AR, Shiraz P, Sidana S, Weng WK, Davis KL, Ramakrishna S, Schultz L, Mullins C, Jacob A, Kirsch I, Feldman SA, Mackall CL, Miklos DB, Muffly L (2021). CD22-directed CAR T-cell therapy induces complete remissions in CD19-directed CAR-refractory large B-cell lymphoma. Blood.

[71] Jia H, Wang Z, Wang Y, Liu Y, Dai H, Tong C, Guo Y, Guo B, Ti D, Han X, Yang Q, Wu Z, Han W (2019). Haploidentical CD19/CD22 bispecific CAR-T cells induced MRD-negative remission in a patient with relapsed and refractory adult B-ALL after haploidentical hematopoietic stem cell transplantation. J Hematol Oncol.

[72] Zeng C, Cheng J, Li T, Huang J, Li C, Jiang L, Wang J, Chen L, Mao X, Zhu L, Lou Y, Zhou J, Zhou X (2020). Efficacy and toxicity for CD22/CD19 chimeric antigen receptor T-cell therapy in patients with relapsed/refractory aggressive B-cell lymphoma involving the gastrointestinal tract. Cytotherapy.

[73] Schneider D, Xiong Y, Wu D, Hu P, Alabanza L, Steimle B, et al. Trispecific CD19-CD20-CD22-targeting duoCAR-T cells eliminate antigen-heterogeneous B cell tumors in preclinical models. Sci Transl Med. 2021;13(586):eabc6401. 10.1126/scitranslmed.abc6401 33762438

[74] Ding S, Mao X, Cao Y, Wang N, Xu H, Zhou J (2020). Targeting CD79b for chimeric antigen receptor T-cell therapy of B-cell lymphomas. Target Oncol.

[75] Scarfò I, Ormhøj M, Frigault M, Castano A, Lorrey S, Bouffard A, van Scoyk A, Rodig S, Shay A, Aster J (2018). Anti-CD37 chimeric antigen receptor T cells are active against B- and T-cell lymphomas. Blood.

[76] Köksal H, Dillard P, Josefsson S, Maggadottir S, Pollmann S, Fåne A, Blaker Y, Beiske K, Huse K, Kolstad A (2019). Preclinical development of CD37CAR T-cell therapy for treatment of B-cell lymphoma. Blood Adv.

[77] Xu-Monette Z, Li L, Byrd J, Jabbar K, Manyam G, Maria de Winde C, van den Brand M, Tzankov A, Visco C, Wang J (2016). Assessment of CD37 B-cell antigen and cell of origin significantly improves risk prediction in diffuse large B-cell lymphoma. Blood.

[78] Goodman A, Patel S, Kurzrock R (2017). PD-1-PD-L1 immune-checkpoint blockade in B-cell lymphomas. Nat Rev Clin Oncol.

[79] Song W, Zhang M (2020). Use of CAR-T cell therapy, PD-1 blockade, and their combination for the treatment of hematological malignancies. Clin Immunol (Orlando, Fla).

[80] Cao Y, Lu W, Sun R, Jin X, Cheng L, He X, Wang L, Yuan T, Lyu C, Zhao M (2019). Anti-CD19 chimeric antigen receptor T cells in combination with Nivolumab are safe and effective against relapsed/refractory B-cell non-hodgkin lymphoma. Front Oncol.

[81] Tao L, Farooq M, Gao Y, Zhang L, Niu C, Ajmal I, et al. CD19-CAR-T Cells Bearing a KIR/PD-1-Based Inhibitory CAR Eradicate CD19HLA-C1 Malignant B Cells While Sparing CD19HLA-C1 Healthy B Cells. Cancers. 2020;12(9):2612. 10.3390/cancers12092612 PMC7564565 32933182

[82] Ebert L, Yu W, Gargett T, Brown M (2018). Logic-gated approaches to extend the utility of chimeric antigen receptor T-cell technology. Biochem Soc Trans.

[83] Han X, Wang Y, Wei J, Han W (2019). Multi-antigen-targeted chimeric antigen receptor T cells for cancer therapy. J Hematol Oncol.

[84] Ayyappan S, Maddocks K (2019). Novel and emerging therapies for B cell lymphoma. J Hematol Oncol.

[85] Yeku O, Brentjens R (2016). Armored CAR T-cells: utilizing cytokines and pro-inflammatory ligands to enhance CAR T-cell anti-tumour efficacy. Biochem Soc Trans.

[86] Huang R, Li X, He Y, Zhu W, Gao L, Liu Y, Gao L, Wen Q, Zhong JF, Zhang C, Zhang X (2020). Recent advances in CAR-T cell engineering. J Hematol Oncol.

[87] Chmielewski M, Abken H (2015). TRUCKs: the fourth generation of CARs. Expert Opin Biol Ther.

[88] Graham C, Jozwik A, Pepper A, Benjamin R. Allogeneic CAR-T Cells: More than Ease of Access? Cells. 2018;7(10):155. 10.3390/cells7100155 PMC6210057 30275435

[89] Yang Y, Jacoby E, Fry T (2015). Challenges and opportunities of allogeneic donor-derived CAR T cells. Curr Opin Hematol.

[90] Liu D, Zhao J, Song Y (2019). Engineering switchable and programmable universal CARs for CAR T therapy. J Hematol Oncol.

